# Inverse Association of Longitudinal Variations in Fat Tissue Radiodensity and Area

**DOI:** 10.3390/diagnostics15131662

**Published:** 2025-06-30

**Authors:** Giulia Besutti, Marta Ottone, Efrem Bonelli, Simone Canovi, Roberto Farì, Francesco Farioli, Annarita Pecchi, Guido Ligabue, Massimo Pellegrini, Pierpaolo Pattacini, Paolo Giorgi Rossi

**Affiliations:** 1 Radiology Unit, Azienda USL—IRCSS di Reggio Emilia, 42123 Reggio Emilia, Italy; 212847@studenti.unimore.it (F.F.); pierpaolo.pattacini@ausl.re.it (P.P.); 2 Department of Medical and Surgical Sciences, University of Modena and Reggio Emilia, 41124 Modena, Italy; annarita.pecchi@unimore.it (A.P.); guido.ligabue@unimore.it (G.L.); 3 Epidemiology Unit, Azienda USL—IRCSS di Reggio Emilia, 42123 Reggio Emilia, Italy; marta.ottone@ausl.re.it (M.O.); paolo.giorgirossi@ausl.re.it (P.G.R.); 4 Department of Clinical Pathology, APSS Trento, 38123 Trento, Italy; efrem.bonelli@apss.tn.it; 5 Clinical Chemistry and Endocrinology Laboratory, Azienda USL—IRCCS di Reggio Emilia, 42123 Reggio Emilia, Italy; simone.canovi@ausl.re.it; 6 Clinical and Experimental Medicine PhD Program, University of Modena and Reggio Emilia, 41124 Modena, Italy; roberto.fari2@gmail.com; 7 Department of Biomedical, Metabolic and Neural Sciences, University of Modena and Reggio Emilia, 41124 Modena, Italy; massimo.pellegrini@unimore.it

**Keywords:** computed tomography, body composition, adipose tissue, radiodensity

## Abstract

Increased CT-derived fat tissue radiodensity has been indicated as a poor prognostic factor in oncological settings, although the reasons are not clear. One hypothesis is that increased radiodensity may reflect the loss of fat droplets within adipocytes, being a proxy of recent weight loss. This study aims to test this hypothesis by evaluating the association between longitudinal variations in fat tissue radiodensity and area in a cohort of COVID-19 patients. Baseline and 2–3-month follow-up chest CT scans of severe COVID-19 pneumonia survivors were retrospectively reviewed to measure subcutaneous, visceral, and intermuscular adipose tissue (SAT, VAT, and IMAT) areas and densities at the T7–T8 vertebrae, and longitudinal variations were computed for each variable. The associations between each compartment area and radiodensity variations (standardized values) were evaluated in univariate linear models and models adjusted by age and sex. A total of 196 COVID-19 survivors with suitable baseline and follow-up CT scans were included (mean age 65 ± 11 years, 62 (31.6%) females, 25% with diabetes and 2.6% with morbid obesity). Longitudinal variation in SAT area was inversely associated with longitudinal variation in SAT radiodensity in univariate models (coeff −0.91, 95%CI = −1.70/−0.12, *p* = 0.02) and after adjustment by age and sex (coeff −0.89, 95%CI = −1.7/−0.09, *p* = 0.03). The effect was similar and stronger for IMAT (coeff −2.1, 95%CI = −3.06/−1.19, *p* < 0.01 in adjusted models), and absent for VAT. Longitudinal variations in subcutaneous and intermuscular adipose tissue areas and densities are inversely associated. Higher adipose tissue radiodensity may be due to decrease in fat area (i.e., weight loss), explaining the poor prognostic effect found in cancer patients.

## 1. Introduction

The influence of body composition on patient outcomes in different kinds of diseases, including infectious diseases and most cancer types, has become apparent in recent decades. Body mass index (BMI) is the first and most basic indicator of body composition. The known “U-shaped” association between BMI and outcomes in cancer patients reflects how both malnourishment and obesity are risk factors, while only having normal BMI or being overweight are protective factors. Among others, this relationship is particularly well described in non-metastatic colorectal cancer, non-small cell lung cancer, and small cell lung cancer [1,2,3].

Furthermore, sudden weight loss can be a sign preceding the diagnosis, associated with poor prognosis in different cancer sites [4]. One of the proposed explanations for the strong relationship between low BMI at cancer diagnosis and poor prognosis is that some patients with low BMI at diagnosis could have experienced recent weight loss due to preexisting illness [5].

Many studies have focused on the poor prognostic impact of low muscle quantity, myosteatosis, and high visceral fat area in oncological and non-oncological settings [6,7,8]. More recently, high fat radiodensity, measured with computed tomography (CT), has also been suggested as a prognostic factor in different cancer types [9,10].

The pathophysiological reasons behind adipose tissue radiodensity variations are unclear. On a systemic level, increasing adipose tissue radiodensity has been described with increasing age or inflammation [11,12]. On a local level, higher fat radiodensity can be the expression of inflamed adipose tissue or the expression of smaller adipocytes [13]. These smaller adipocytes may be smaller per se, reflecting a healthier fat tissue [14], or may be the result of shrinkage and fibrosis, possibly consequent to a decrease in the fat volume without a reduction in adipocytes, associated with recent weight loss [15]. In this regard, there is the possibility that the poor prognostic effect of higher fat radiodensity that has been shown in cancer patients could only be a proxy of weight loss before diagnosis, due to pre-existing illness.

CT scans acquired for various clinical reasons, e.g., cancer staging, allow the collection of information on body composition, providing an opportunistic screening of muscle and fat parameters [16].

In a cohort of severe COVID-19 pneumonia patients who were prospectively followed with chest CT for the evaluation of lung damage, we previously reported body composition changes from baseline to 2–3-month follow-up CT scan [17]. Among these changes, muscle and fat tissue loss was common and associated with higher inflammatory burden, which is also associated with worse lung damage [18]. Using this cohort, we tested the hypothesis of a relationship between fat loss and increase in fat tissue radiodensity. This population was selected based on the availability of two consecutive CT body composition assessments and the presence of a substantial proportion of patients who experienced weight loss between the two CT scans. To test the hypothesis, we measured the association between changes in fat area, as a proxy of overall fat volume [16], and changes in fat radiodensity in different fat compartment areas, including subcutaneous, visceral, and intramuscular fat.

## 2. Materials and Methods

### 2.1. Study Design and Ethics

This study is a secondary analysis of a previously published single-center retrospective cohort [17]. None of the primary dependent variables presented in this manuscript, namely, fat density measurements, have been analyzed or reported in prior publications from this cohort.

In our institution, during the first wave of the COVID-19 pandemic, all suspected COVID-19 pneumonia patients underwent a baseline CT scan at the Emergency Department. Then, a routine 2–3-month follow-up CT scan was offered to survivors who had been hospitalized for severe pneumonia with clinical–radiological features that have been previously reported [19].

The study was approved by the Area Vasta Emilia Nord (AVEN) Ethics Committee (protocol number 855/2020/OSS/AUSLRE, date of approval 28 July 2020). The research was performed in accordance with the Declaration of Helsinki. Given the retrospective nature of the data collection, the Ethics Committee authorized the use of a patient’s data without his/her informed consent if all reasonable efforts had been made to contact the patient to obtain it.

### 2.2. Study Population

All COVID-19 survivors who underwent a routine 2–3-month follow-up CT scan were considered eligible for the study. Patients with unsuitable CT scans for body composition measurements due to artifacts, including examinations performed with arms alongside the body, were excluded. This study population is a subgroup of the previously reported study describing body composition variations from baseline to follow-up CT scan [17]. In this previous study, fat tissue radiodensity in different fat compartments was not analyzed.

### 2.3. Data Collection

Methods for data collection have been previously described in detail [17]. For this study, only sex, age, and comorbidities were considered. Moreover, the occurrence of acute kidney failure during hospital stay for COVID-19 pneumonia was collected through a review of medical records.

### 2.4. CT Retrospective Analysis

Body composition CT assessment on both baseline and 2–3-month follow-up CT images was retrospectively conducted by a single trained image analyzer supervised by a senior radiologist, both blinded to clinical data and outcomes, by using OSIRIX-Lite software V5.0 (Pixmeo SARL, Bernex, Switzerland).

**Figure 1:**
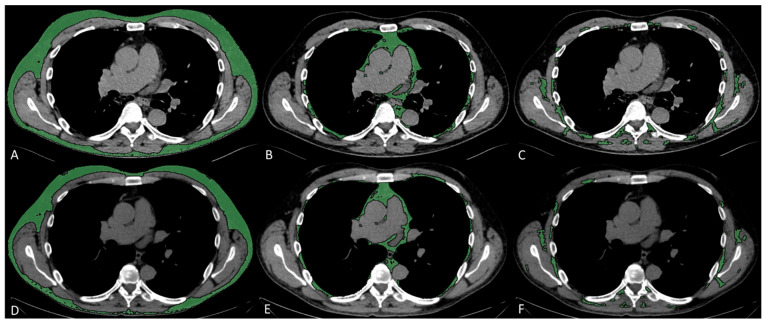
Fat characteristic measurement. Representative CT images analyzed through semiautomated segmentation of different fat compartments. The measurement of baseline SAT (**A**), VAT (**B**), and IMAT (**C**), and 2–3-month follow-up SAT (**D**), VAT (**E**), and IMAT (**F**), showed a decrease in SAT area from 127 to 107 cm^2^ and an increase in SAT density from −104 to −90 HU, a decrease in VAT area from 36 to 31 cm^2^ and an increase in VAT density from −89 to −80 HU, a decrease in IMAT area from 21 to 13 cm^2^, and an increase in IMAT density from −73 to −61 HU.

Subcutaneous, visceral, and intermuscular adipose tissue areas (SAT, VAT, and IMAT) were measured on a single slice at the level of the seventh to eighth thoracic vertebrae, by applying a radiodensity range from −190 to −30 HU, through autosegmentation and manual contour correction when necessary. These measurements conducted at T7–T8 showed good reproducibility [17,20], and were previously shown to be representative of total chest fat and moderately associated with abdominal fat [21]. For each fat compartment, mean radiodensity (HU) was also collected. Representative images of fat measurements and respective longitudinal changes are reported in Figure 1.

### 2.5. Statistical Analyses

Continuous variables were reported as mean (standard deviation—SD) and categorical variables as numbers and percentages (%).

The correlation between each fat compartment area and radiodensity at each timepoint was preliminarily assessed. After checking the distribution for normality using the Shapiro–Wilk test and finding a non-normal distribution, Spearman’s correlation coefficients were used (Supplementary Table S1). The association between area variation from baseline to 2–3-month CT scan and radiodensity variation from baseline to 2–3-month CT scan for each fat compartment (SAT, VAT, IMAT) were estimated by Spearman’s correlation coefficient and by linear regression coefficients in both univariate models and models adjusted by age and sex. In linear regression analyses, fat tissue areas were used as standardized variables. A sensitivity analysis was conducted by adjusting the models for SAT area changes, considered a distal determinant of VAT and IMAT area. A second sensitivity analysis was performed by restricting models to patients who did not experience acute kidney failure during COVID-19 pneumonia. In fact, acute renal failure, by inducing fluid retention and fat tissue edema, could be a confounding factor. Finally, stratified models for males and females are reported.

*p*-values are reported as continuous measures, and no significance threshold was set. All statistical analyses were conducted using Stata/IC 16.1 statistical software (Stata Corp., College Station, TX, USA).

## 3. Results

### 3.1. Study Population

The flow chart for patient inclusion has been previously reported (14). After exclusion of 12 patients with CT scans unsuitable for fat area and radiodensity evaluation, 196 patients with two timepoints (baseline and 2–3-months follow-up) with suitable CT scans were included.

Mean age was 65 (±11) years and 62 (31.6%) were females; comorbidities are reported in Table 1.

### 3.2. Fat Areas and Densities

Mean fat areas for visceral, subcutaneous, and intermuscular compartments, and respective densities, both from baseline and 2–3-month follow-up CT scans, are reported in Table 2. In the subgroup of patients with available BMI, baseline fat areas were significantly associated with BMI, especially SAT (Supplementary Table S2). At each timepoint and for each compartment, fat areas were inversely associated with fat densities (*p* < 0.001), with stronger associations for SAT (Spearman’s rho −0.7) (Table 3 and Supplementary Figure S1).

### 3.3. Fat Area and Radiodensity Variations

**Figure 2:**
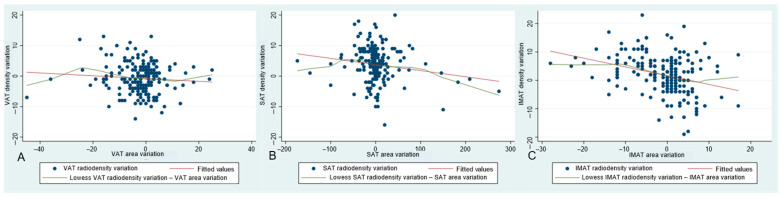
Graphs representing associations between area variation and density variation from baseline to 2–3-month follow-up CT scan, for visceral adipose tissue (VAT) (**A**), subcutaneous adipose tissue (SAT) (**B**), and intermuscular adipose tissue (IMAT) (**C**), respectively.

Changes in areas and densities from baseline to 2–3-month follow-up were inversely associated for SAT and IMAT, but not for VAT (Supplementary Table S3 and Figure 2).

In univariate models for fat radiodensity changes and in models adjusted by age and sex, SAT and IMAT area changes were inversely associated with respective radiodensity changes, while VAT area change was not associated with VAT radiodensity changes (Table 4). The associations for IMAT and VAT did not change adjusting for SAT area changes (Supplementary Table S4).

When excluding 10 patients who experienced acute renal failure during COVID-19 disease, the results of univariate and adjusted models were unchanged (Supplementary Table S5).

In sex-stratified models adjusted by age, no substantial variations were found for VAT and IMAT changes, while for SAT the association was appreciable in female patients only (coeff −1.00, 95%CI −1.94–−0.07; *p* = 0.036) (Supplementary Table S6).

## 4. Discussion

In this retrospective study evaluating fat compartments at baseline and after 2–3 months from diagnosis in severe COVID-19 pneumonia survivors, changes in fat radiodensity in the subcutaneous and intermuscular compartment were inversely associated with variations in respective fat areas. For subcutaneous fat, the relationship was appreciable in female patients only. For the visceral compartment, even if the relationship had the same inverse direction, the association was compatible with random fluctuations.

Cross-sectional fat areas at the abdominal level (L3) have been shown to be highly associated with total fat volume [19]. In this study, fat areas were evaluated at the thoracic level; however, in previous studies conducted on the same population [20], a similar high association was found between thoracic fat areas and BMI. Thus, the reduction in fat area can be considered one of the expressions of weight loss.

With these premises, the finding of an inverse relationship between variations in fat areas and densities confirms the hypothesis that a higher fat radiodensity may reflect recent weight loss. This study was conducted on a cohort of COVID-19 survivors for opportunistic reasons, as we already knew that fat loss in this cohort was common and that CT scans had been routinely prospectively performed at baseline and 2–3-month follow-up [17]. Although this population does not allow for direct generalization to oncologic settings, it offered a suitable model to test our hypothesis. The 1-point average decrease in BMI that was registered in this population [17] reflects a milder weight loss than that commonly seen in cancer patients in advanced disease stage. However, despite conveying this inverse relationship between changes in fat areas and densities to the cancer setting remaining speculative, it is sufficient to consider it as a potential source of biases in studies investigating the association between fat density and cancer outcomes. In fact, weight and fat loss prior to cancer diagnosis are common and represent poor prognostic factors for some cancers [6,9,10].

Both subcutaneous and visceral adipose tissue increased radiodensity have been associated with poor prognosis in cancer patients; however, SAT radiodensity has demonstrated more robust results [9,22,23]. This seems to be consistent with our finding that increase in fat radiodensity reflects decrease in fat areas in the subcutaneous more than in the visceral compartment. A possible explanation for this difference among fat compartments may be the higher influence of several systemic and local factors on VAT radiodensity. In fact, the visceral compartment is more involved in metabolic processes, energy storage, and systemic inflammation [24]. This is especially demonstrated for abdominal VAT, but also thoracic and epicardial visceral fat depots are involved in inflammatory processes, as well as being associated with abdominal VAT [25]. SAT radiodensity, on the other hand, may be less influenced by the aforementioned factors, and hence more sensitive to weight loss.

The high proportion of missing data on weight and BMI should be mentioned as a major limitation of the present study. While we interpreted reductions in fat area as a proxy for weight loss, this remains speculative in the absence of serial body weight measurements. Another limitation of our study was the lack of data on systemic inflammation; however, we can hypothesize that higher inflammation leads to an increase in fat radiodensity, while in this cohort the inflammatory burden decreased from baseline to follow-up [18], and at the same time fat radiodensity increased. Hence, we cannot envisage a substantial change in results even considering information on inflammatory status. Moreover, we could not stratify for those who received parenteral nutrition or other nutritional support to see whether the association changes accordingly. Finally, some generalizability concerns may be raised by the selection of hospitalized, hence seriously ill, COVID-19 patients, and confirmation is needed in other patient settings.

In conclusion, variations in fat radiodensity in the subcutaneous and intermuscular compartments are inversely associated with variations in fat area, seemingly confirming the hypothesis that a higher fat radiodensity may reflect recent weight loss. This observation suggests that recent weight loss can be a potential confounder in studies investigating the association between fat density and cancer outcomes. Given that weight loss before cancer diagnosis is common and it is associated with poor prognosis, studies investigating any causal link between fat density and cancer prognosis should try to exclude this possible confounding effect.

## Figures and Tables

**Table 1:** Demographic and clinical characteristics of the included patients.

		Missing	Study Population (*n* = 196)
**Age (Years); Mean (SD)**			65 (11.0)
Sex; *n* (%)	Females		62 (31.6%)
	Males		134 (68.4%)
Smoking habit	Never	6	160 (84.2%)
	Previous		27 (14.2%)
	Current		3 (1.6%)
Comorbidities; *n* (%)		6	
	COPD		8 (4.2%)
	Asthma		9 (4.7%)
	Cardiovascular diseases		47 (24.7%)
	Previous cancer		20 (10.5%)
	Diabetes		44 (23.2%)
	Hypertension		114 (60.0%)
	Chronic kidney failure		7 (3.7%)
	Cerebrovascular disease		9 (4.7%)
	Liver diseases		6 (3.2%)
Baseline BMI (kg/m^2^); mean (SD)		138	30.2 (6.5)
Acute kidney failure during COVID-19, *n* (%)			10 (5,1%)

SD, Standard Deviation; COPD, Chronic Obstructive Pulmonary Disease; BMI, Body Mass Index.

**Table 2:** Fat areas and densities.

	Missing	Baseline (*n* = 196)	Follow-Up (*n* = 196)
VAT area (cm^2^), mean (SD)	2	40.9 (19.4)	38.6 (18.4)
VAT radiodensity (HU), mean (SD)	-	−83.4 (5.7)	−84.1 (5.1)
SAT area (cm^2^), mean (SD)	4	204.1 (118.1)	204.8 (129.4)
SAT radiodensity (HU), mean (SD)	1	−96.9 (8.0)	−93.1 (8.7)
IMAT area (cm^2^), mean (SD)	3	32.7 (16.9)	31.4 (16.1)
IMAT radiodensity (HU), mean (SD)	-	−70.3 (7.8)	−68.3 (7.7)

VAT, Visceral Adipose Tissue; SAT, Subcutaneous Adipose Tissue; IMAT, Intermuscular Adipose Tissue; SD, Standard Deviation; HU, Hounsfield Units.

**Table 3:** Associations of fat areas and densities.

	Coeff (Spearman’s Rho)	*p*-Value
Baseline VAT area and radiodensity	−0.52	<0.001
Baseline SAT area and radiodensity	−0.68	<0.001
Baseline IMAT area and radiodensity	−0.57	<0.001
Follow-up VAT area and radiodensity	−0.44	<0.001
Follow-up SAT area and radiodensity	−0.73	<0.001
Follow-up IMAT area and radiodensity	−0.56	<0.001

VAT, Visceral Adipose Tissue; SAT, Subcutaneous Adipose Tissue; IMAT, Intermuscular Adipose Tissue.

**Table 4:** Linear models for fat radiodensity variations. For each model, the dependent variable is the fat radiodensity variation of the same district.

		Univariate Models			Models Adjusted by Age and Sex		
	*n*	Coeff	95% CI	*p*	Coeff	95% CI	*p*
VAT area variation (STD)	194	−0.38	−1.07; 0.30	*0.27*	−0.33	−1.02; 0.37	*0.35*
SAT area variation (STD)	190	−0.91	−1.70; −0.12	*0.02*	−0.89	−1.70; −0.09	*0.03*
IMAT area variation (STD)	192	−2.12	−3.02; −1.21	*<0.001*	−2.12	−3.06; −1.19	*<0.001*

STD, standardized value; CI, confidence interval; VAT, Visceral Adipose Tissue; SAT, Subcutaneous Adipose Tissue; IMAT, Intermuscular Adipose Tissue.

## Data Availability

Participant data that underlie the results reported in this manuscript will be shared after de-identification, beginning 6 months and ending at least 7 years after article publication, to researchers who provide a methodologically sound proposal with objectives consistent with those of the original study. Proposals and data access requests should be directed to the Area Vasta Emilia Nord (AVEN) Ethics Committee at CEReggioemilia@ausl.re.it as well as to the Authors at the Epidemiology Unit of AUSL–IRCCS di Reggio Emilia at info.epi@ausl.re.it, who are the data guardians. To gain access, data requestors will need to sign a data access agreement.

## Supplementary Materials

The following supporting information can be downloaded at: https://www.mdpi.com/article/10.3390/diagnostics15131662/s1, Figure S1. Correlation between fat areas and radiodensities at each timepoint; Table S1: Shapiro–Wilk test for normality of the distributions of the fat areas and radiodensities, at the two timepoints; Table S2. Pearson’s correlation coefficients between baseline BMI and fat areas, *p*-values, and number of valid observations. Table S3: Association between area variations and radiodensity variations in each compartment; Table S4: Linear models for fat radiodensity changes. Table S5: Linear models for fat radiodensity variations after excluding 10 patients who experienced acute renal failure during COVID-19. Table S6: Linear models stratified by sex and adjusted by age for fat radiodensity variations.

## Abbreviations

The following abbreviations are used in this manuscript:

AVEN	Area Vasta Emilia Nord
BMI	Body Mass Index
CI	Confidence Interval
COPD	Chronic Obstructive Pulmonary Disease
CT	Computed Tomography
HU	Hounsfield Unit
IMAT	Intermuscular Adipose Tissue
SAT	Subcutaneous Adipose Tissue
SD	Standard Deviation
VAT	Visceral Adipose Tissue
