# Supplementary material for: Inverse Association of Longitudinal Variations in Fat Tissue Radiodensity and Area
Source: Diagnostics (Basel). 2025 Jun 30;15(13):1662. doi: 10.3390/diagnostics15131662 (PMC12249357; doi:10.3390/diagnostics15131662)
Supplement: Supplementary file 1 [file diagnostics-15-01662-s001.zip › diagnostics-3448274-supplementary.pdf]

Supplementary Table S1. Shapiro–Wilk test for normality of the distributions of the fat areas and radiodensities, at the two timepoints.

Variable	Obs	W	V	z	Prob > z
VAT area t1	194	0.93467	9.492	5.170	0.00000
VAT density t1	196	0.99244	1.108	0.236	0.40664
SAT area t1	193	0.89503	15.182	6.247	0.00000
SAT density t1	196	0.96274	5.461	3.902	0.00005
IMAT area t1	193	0.91829	11.818	5.672	0.00000
IMAT density t1	196	0.98324	2.456	2.065	0.01944
VAT area t2	195	0.92971	10.258	5.350	0.00000
VAT density t2	196	0.99735	0.388	-2.177	0.98526
SAT area t2	192	0.87145	18.509	6.701	0.00000
SAT density t2	195	0.97206	4.078	3.230	0.00062
IMAT area t2	193	0.92483	10.873	5.480	0.00000
IMAT density t2	196	0.97384	3.835	3.090	0.00100

VAT, Visceral Adipose Tissue; SAT, Subcutaneous Adipose Tissue; IMAT, Intermuscolar Adipose Tissue; Obs, observations.

Supplementary Table S2. Pearson's correlation coefficients between baseline BMI and fat areas, p-values, and number of valid observations.

	BMI	VAT	SAT	IMAT
BMI	1.000<br><br>obs 58			
VAT	0.325<br>p 0.013<br>obs 58	1.000<br><br>obs 194		
SAT	0.770<br>p<0.001<br>obs 58	0.239<br>p<0.001<br>obs 193	1.000<br><br>obs 193	
IMAT	0.527<br>p<0.001<br>obs 58	0.648<br>p<0.001<br>obs 193	0.503<br>p<0.001<br>obs 193	1.000<br><br>obs 193

VAT, Visceral Adipose Tissue; SAT, Subcutaneous Adipose Tissue; IMAT, Intermuscolar Adipose Tissue; Obs, observations.

Supplementary Figure S1: Correlation between fat areas and radiodensities at each timepoint.

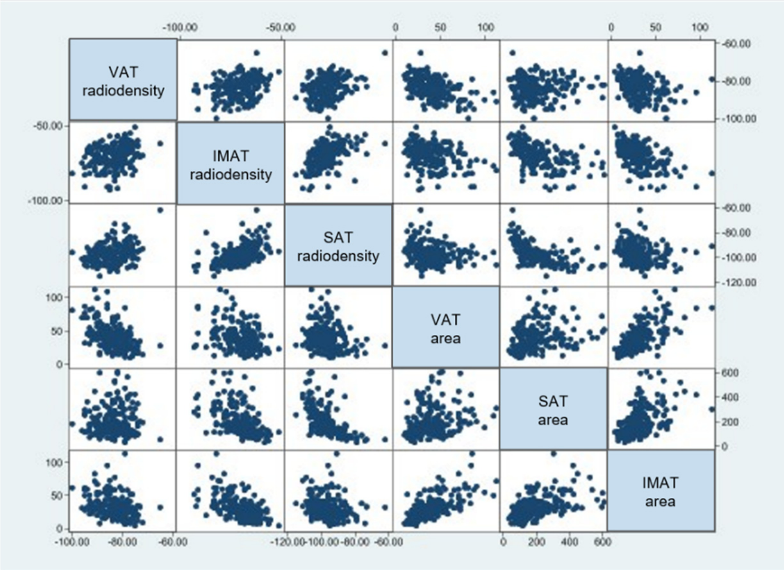

A

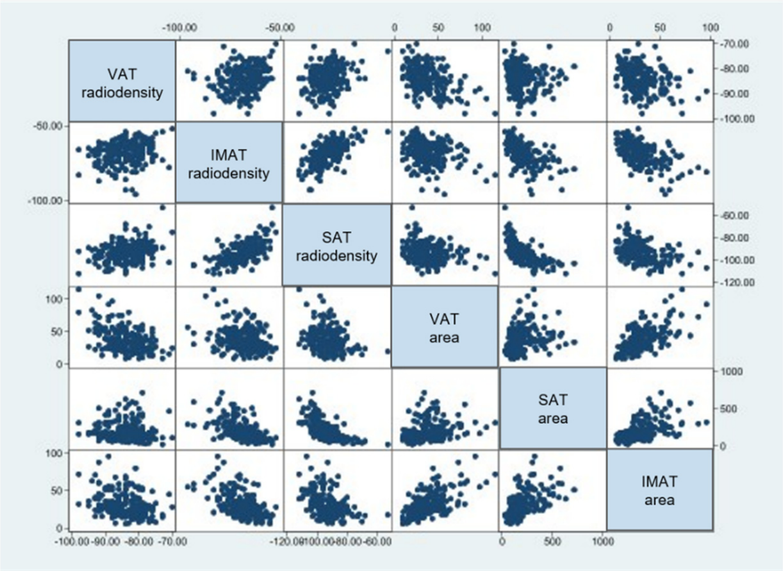

B

Supplementary Figure S1: Graphs representing linear associations between fat areas and radiodensity at baseline CT scan (A) and at 2–3-month follow-up CT scan (B).

Supplementary Table S3. Association between area variations and radiodensity variations in each compartment.

	**Coeff (Spearman's rho)**	***p*-Value**
VAT area and radiodensity variations	-0.040	0.589
SAT area and radiodensity variations	-0.151	0.039
IMAT area and radiodensity variations	-0.303	<0.001

VAT, Visceral Adipose Tissue; SAT, Subcutaneous Adipose Tissue; IMAT, Intermuscolar Adipose Tissue.

Supplementary Table S4. Linear models for fat radiodensity changes. The respective dependent variables are VAT density change for the coefficient reported for VAT area change and IMAT density change for the coefficient reported for IMAT area change. Models adjusted by age, sex, and SAT area change.

Variable	n	Coeff.	95% CI	*p*
VAT area change (STD)	194	-0.33	-1.03 to 0.37	0.35
IMAT area change (STD)	192	-2.19	-3.12 to -1.26	<0.001

STD, standardized value; CI, confidence interval; VAT, Visceral Adipose Tissue; SAT, Subcutaneous Adipose Tissue; IMAT, Intermuscolar Adipose Tissue.

Supplementary Table S5: Linear models for fat radiodensity variations after excluding 10 patients who experienced acute renal failure during COVID-19.

		**Univariate models**			**Models adjusted by age and sex**		
	**n**	**coeff**	**95% CI**	***p***	**coeff**	**95% CI**	***p***
VAT area variation (STD)	184	-0.44	-1.14; 0.26	*0.21*	-0.42	-1.13; 0.29	*0.25*
SAT area variation (STD)	181	-1.01	-1.85; -0.17	*0.02*	-0.99	-1.84; -0.14	*0.02*
IMAT area variation (STD)	183	-2.11	-3.03; -1.18	*<0.001*	-2.10	-3.06; -1.15	*<0.001*

STD, standardized value; CI, confidence interval; VAT, Visceral Adipose Tissue; SAT, Subcutaneous Adipose Tissue; IMAT, Intermuscolar Adipose Tissue.

Supplementary Table S6: Linear models stratified by sex and adjusted by age for fat radiodensity variations.

	Models adjusted by age								
	Females				Males				*p interaction*
	n	coeff	95% CI	*p*	n	coeff	95% CI	*p*	
VAT area variation (STD)	61	-0.59	-2,13; 0.94	*0.44*	133	-0.25	-1.02; 0.53	*0.53*	*0.756*
SAT area variation (STD)	59	-1,00	-1.94; -0.70	*0.04*	131	-0.09	-1.73; 1,55	*0.92*	*0.268*
IMAT area variation (STD)	60	-2.10	-3.90; -0,31	*0.02*	132	-2.13	-3.24; -1.02	*<0.001*	*0.986*

STD, standardized value; CI, confidence interval; VAT, Visceral Adipose Tissue; SAT, Subcutaneous Adipose Tissue; IMAT, Intermuscolar Adipose Tissue
